# Single‐cell profiling reveals the metastasis‐associated immune signature of hepatocellular carcinoma

**DOI:** 10.1002/iid3.1264

**Published:** 2024-05-23

**Authors:** Deyuan Zhong, Ying Shi, Wenzhe Ma, Yuxin Liang, Hanjie Liu, Yingying Qin, Lu Zhang, Qinyan Yang, Xiaolun Huang, Yuanjun Lu, Jin Shang

**Affiliations:** ^1^ Liver Transplantation Center and HBP Surgery, Sichuan Clinical Research Center for Cancer, Sichuan Cancer Hospital & Institute, Sichuan Cancer Center Affiliated Cancer Hospital of University of Electronic Science and Technology of China Chengdu China; ^2^ School of Medicine University of Electronic Science and Technology of China Chengdu Sichuan China; ^3^ State Key Laboratory of Quality Research in Chinese Medicine Macau University of Science and Technology Macau SAR China; ^4^ Sichuan Clinical Research Center for Cancer, Sichuan Cancer Hospital & Institute, Sichuan Cancer Center Affiliated Cancer Hospital of University of Electronic Science and Technology of China Chengdu China

**Keywords:** dendritic cells, hepatocellular carcinoma, metastasis, single cell RNA sequencing, Treg cells, tumor immune microenvironment

## Abstract

**Aim:**

Metastasis is the leading cause of mortality in hepatocellular carcinoma (HCC). The metastasis‐associated immune signature in HCC is worth exploring.

**Methods:**

Bioinformatic analysis was conducted based on the single‐cell transcriptome data derived from HCC patients in different stages. Cellular composition, pseudotime state transition, and cell–cell interaction were further analyzed and verified.

**Results:**

Generally, HCC with metastasis exhibited suppressive immune microenvironment, while HCC without metastasis exhibited active immune microenvironment. Concretely, effector regulatory T cells (eTregs) were found to be enriched in HCC with metastasis. PHLDA1 was identified as one of exhaustion‐specific genes and verified to be associated with worse prognosis in HCC patients. Moreover, A novel cluster of CCR7^+^ dendritic cells (DCs) was identified with high expression of maturation and migration marker genes. Pseudotime analysis showed that inhibition of differentiation occurred in CCR7^+^ DCs rather than cDC1 in HCC with metastasis. Furthermore, interaction analysis showed that the reduction of CCR7^+^ DCs lead to impaired CCR7/CCL19 interaction in HCC with metastasis.

**Conclusions:**

HCC with metastasis exhibited upregulation of exhaustion‐specific genes of eTregs and inhibition of CCL signal of a novel DC cluster, which added new dimensions to the immune landscape and provided new immune therapeutic targets in advanced HCC.

## Graphical abstract

Bioinformatic analysis based on single‐cell transcriptome data was conducted to explore the landscape of the metastasis‐related signature of immune cells. Impaired maturation of dendritic cells and enhanced immunosuppression of Treg cells were the main metastasis‐related features of HCC.

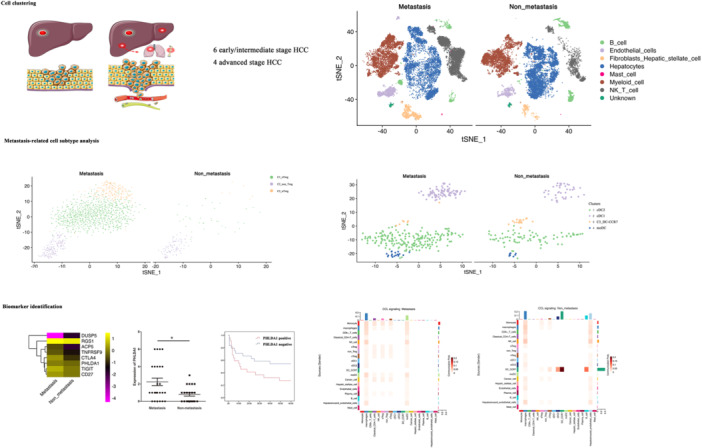

## 1 INTRODUCTION

Improved prognosis has been achieved in many neoplastic diseases.^1^ However, metastasis, a distinct hallmark of cancer, remains the leading cause of the risen or stable mortality in certain tumor diseases.^2^ Specifically, Hepatocellular carcinoma (HCC) was estimated to be the sixth most commonly diagnosed cancer and the third leading cause of cancer death worldwide.^3^ Overall survival of advanced HCC with metastasis is only about 11–13 months.^4^, ^5^ Metastasis remains the major obstacle of the prognosis of HCC patients.

The tumor microenvironment, including immune cells, extracellular matrix, inflammatory factors, and others, influences the tumor progression.^6^, ^7^ Effective antitumor immunity could prevent tumorigenesis, post‐resection recurrence and metastasis. However, immunosuppressive cells included myeloid‐derived suppressor cells (MDSCs) and regulatory T cells (Tregs)^8^, ^9^ could also contribute to tumor growth, progression, and metastasis.^10^ Recently, the landscape of infiltrating immune cells in HCC has been gradually revealed. Specific immune cells such as exhausted CD8^+^ T cells and Tregs were enriched in HCC.^11^ Dendritic cells (DCs) played a critical role in immune cross‐talk, while tumor‐associated macrophages (TAMs) were associated with poor prognosis.^12^ Generally, the suppressive immune microenvironment facilitates the carcinogenesis of HCC and has become an obstacle in HCC systemic therapy.

Immune checkpoint inhibitors (ICIs) are the most promising therapy in HCC,^13^, ^14^, ^15^ while overcoming ICIs resistance remains a challenge. Metastasis‐related processes such as accumulation of extracellular matrix, abnormal angiogenesis and oxygen supplement could inhibit the immune infiltrating and influence the efficacy of ICIs.^16^ Thus, it's meaningful to figure out the HCC‐metastasis immune signature. Notably, HCC metastasis‐related immune microenvironment was reported to be reshaped by TAMs and specific subpopulations of malignant hepatocytes.^17^ However, it's lack of detailed description of the landscape of metastasis‐associated immune cells involved in ICIs resistance‐related processes such as T cell exhaustion, restricted antigen‐presenting, etc. Therefore, the present study analyzed single‐cell transcriptome data derived from HCC in different stages, hoping to provide new insights into immunotherapy and help overcome the ICIs resistance in advanced HCC.

## 2 METHODS

### 2.1 Data source

The scRNA‐seq files (raw unique molecular identifier [UMI]) counts based on 10× Genomics technology) from 10 HCC tissues were accessed from GSE149614. Tumor‐node‐metastasis staging system was used. Six patients were in the early/intermediate stage without metastasis. Four patients were in advanced with metastasis.

### 2.2 Single‐cell RNA‐seq data preprocessing

The Cell Ranger software pipeline (version 5.0.0) provided by 10× Genomics was used to demultiplex cellular barcodes and map reads to the genome and transcriptome.

### 2.3 Quality control

R package Seurat (version 3.1.1) was applied for processing the UMI count matrix20. Detected gene numbers less than 200, UMI less than 1000 and log10 Genes Per UMI less than 0.7 were ruled out. Low‐quality cells where >10% of the counts belonged to mitochondrial genes and >5% of the counts belonged to hemoglobin genes were discarded. Totally 34,414 single cells were included in downstream analysis.

### 2.4 Marker genes and cell subsets identifying

FindAllMarkers function was used to identify marker genes of each cluster. R package SingleR (version 1.4.1), a novel computational method, was applied for unbiased cell type recognition of scRNA‐seq, with the reference transcriptomic datasets Human Primary Cell Atlas to infer the cell of origin of each of the single cells independently and identify cell types. B cells were characterized by high expression of CD19, CD20, and MS4A1. NK cells were characterized by high expression of CD16, CD56, and NCAM1, and DCs were characterized by high expression of HLA‐DR. Macrophage cells were characterized by high expression of CD11b, CD68, and ITGAM. Tregs were characterized by high expression of CD25, CD4, IL2RA, and FOXP3. MDSCs were characterized by high expression of CD11b, ITGAM, CD14, CD15, and FUT4. Mast cells were characterized by high expression of CD11b, ITGAM, CD117, KIT, CD203c, and ENPP3. Neutrophil cells were characterized by high expression of CD11b, CD63, CD66b, and CEACAM8. Endothelial cells were characterized by high expression of CLEC4G, EHD3, STAB2, PTPRB, AQP1, CDH5, FLT1, and CD34. T cells were characterized by high expression of CD3G, CD3E, CD3D, and THY1. Hepatocytes were characterized by high expression of ALB, APOC3, APOB, APOA1, HPR, SEC. 16B, BCHE, and others. Hepatic stellate cells were characterized by high expression of DCN, ACTA2, and COL1A1.

### 2.5 Differential expressed genes (DEGs) analysis

*p*‐Value < .05 and log 2 foldchange > 0.58 was set as the threshold for significantly differential genes expression. GO and KEGG pathway analysis of DEGs were respectively performed using R based on the hypergeometric distribution.

### 2.6 Preparation of peripheral blood mononuclear cells (PBMCs) from HCC patients

Peripheral blood mononuclear cells (PBMCs) were obtained from three HCC patients. Written informed consents were signed from each patient in accordance with the Declaration of Helsinki. PBMCs were isolated using Human Lymphocyte separation medium Ficoll (GE lifesciences) and cultured in RPMI‐1640 medium containing 10% fetal bovine serum (FBS; Gibco; Thermo Fisher Scientific). 100 U/mL penicillin and 100 ng/mL streptomycin were added to the medium.

### 2.7 DCs separation and maturation

Monocytes were isolated by CD14 MicroBeads (Miltenyi) using MACS multistand and MidiMACS separator (Miltenyi). Expression of CD14 was analyzed by flow cytometry and purity of the CD14+ cells was >95%. Purified CD14+ cells were then cultured in RPMI‐1640 medium containing 10% FBS, 100 U/ml penicillin, and 100 ng/mL streptomycin at the concentration of 1%, supplemented with 40 ng/ml GM‐CSF (Novoprotein) and 20 ng/mL IL‐4 (Novoprotein). Cells were incubated at 37°C and 5% CO_2_ for 7 days. The fresh medium was replaced gently every 3 days. DCs were matured for another 48 h by adding 10 ng/mL TNF‐α, 1000 U/mL IL‐6, and 1 mg/mL PGE2 (both from Novoprotein).

### 2.8 Flow cytometry

Tumor‐infiltrating lymphocytes were isolated by CD45 McroBeads using MACS multistand and MidiMACS separator (both from Miltenyi). Anti‐human HLA‐DR (Pacific Blue), CD80 (FITC), CD86 (PE), CCR7(BV 650 TM), and Lin (BV 510TM) were purchased by CST, USA, and used for identifying maturation of DCs.

### 2.9 Immunohistochemical staining (IHC)

HCC tissues derived from 46 patients were fixed in 10% formalin for 48 h and embedded in paraffin to make specimens. The expression of proteins was investigated by IHC. In IHC, we incubated the specimens with 1:150 rabbit monoclonal to PHLDA1 (Abcam) overnight at 4°C. IHC results were evaluated by calculating the percentage of positive cells and the staining concentration in five random fields of views. Every assessment was carried out by two separate professional pathologists. The percentage of positive cells and mesenchymal area was scored as follows: 0 = 0%–10%; 1 = 11%–25%; 2 = 26%–50%; 3 = 51%–75%; 4 = 76%–100%. The intensity of positive staining was scored as follows: 0 = no staining; 1 = low staining; 2 = moderate staining; 3 = strong staining. Experiments were performed in triplicate.

### 2.10 Statistical analysis

Statistical analysis was performed with SPSS software (version 20.00), and the statistical graphs were drawn by the software GraphPadPrisms v5.0. Measurement data conforming to normal distribution were expressed by mean and standard deviation, and the two‐sample *t*‐test was conducted to compare the differences between groups. Survival analysis was analyzed based on overall survival and statistical significance was evaluated by Kaplan–Meier analysis and log‐rank test. *p* < .05 was considered as statistically significant.

## 3 RESULTS

### 3.1 Immune landscape of HCC with different stages by single‐cell RNA‐seq profiling

**Figure 1:**
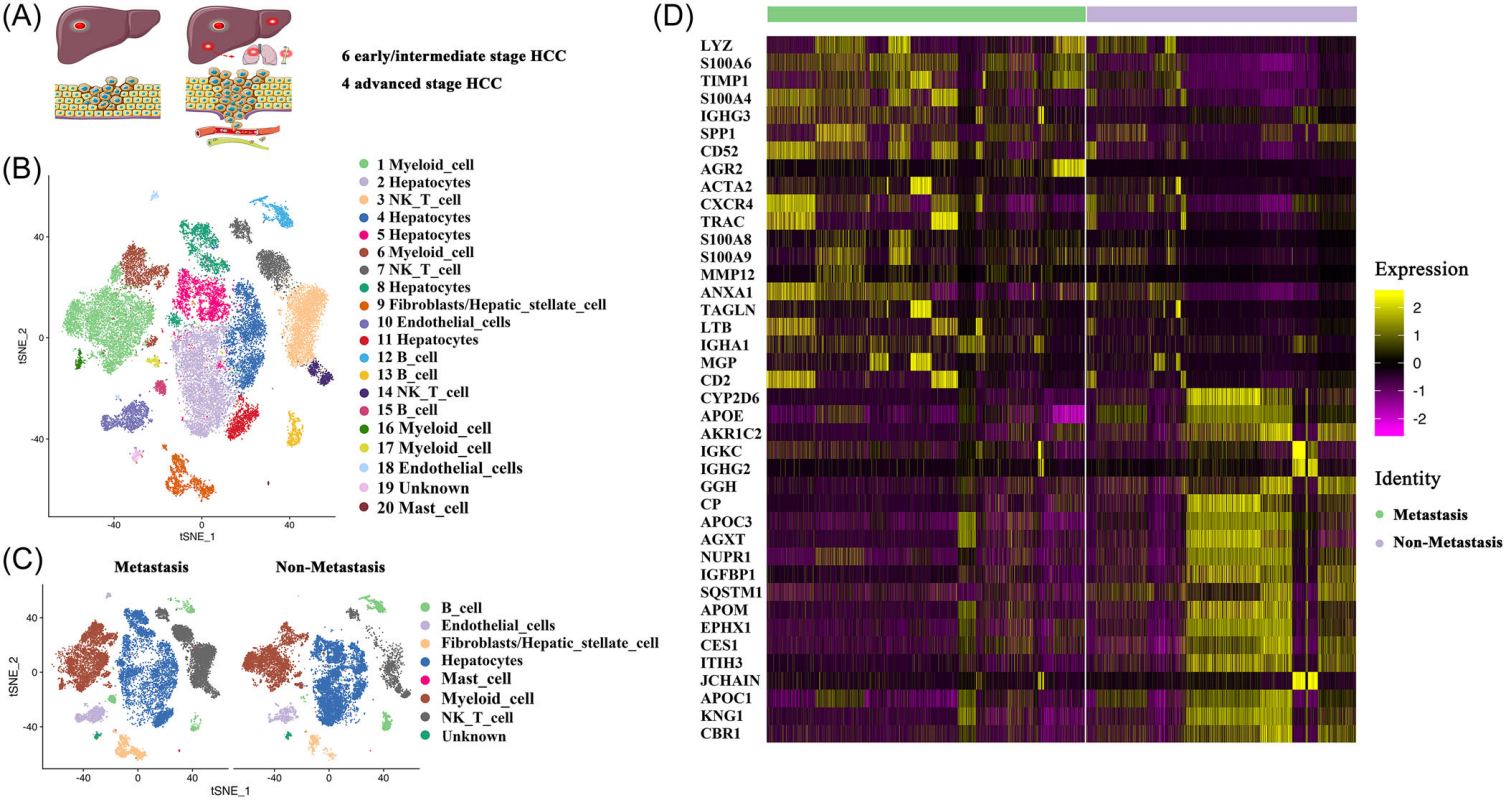
Landscape of tumor‐infiltrating immune cells in hepatocellular carcinoma (HCC) at different stages. (A) Single‐cell RNA‐seq data derived from six HCC patients at early/intermediate stages and four HCC patients at advanced stages were analyzed. (B) Twenty major cell clusters were identified. (C) Composition of cell types in metastasis and non‐metastasis groups. (D) Heatmap of DEGs between metastasis and non‐metastasis group. Differentially expressed genes, DEGs.

We analyzed single‐cell RNA‐seq data of 10 HCC tissues derived from the GEO database GSE149614. Six HCC patients were at early/intermediate stages with no evidence of metastasis and divided into the non‐metastasis group. Meanwhile, four patients were at advanced stages with evidence of metastasis and divided into the metastasis group (Figure 1A). In total, we obtained single‐cell RNA‐seq data from 34,414 single cells after preprocessing and quality control.

Clustering analysis identified 20 distinct cell clusters, which could be classified into major cell types, including B cells, endothelial cells, fibroblast/hepatic stellate cells, hepatocytes, mast cells, myeloid cells, and NK‐T cells (Figure 1B). Unknown cell cluster exhibited similar characteristics with fibroblast and hepatic stellate cells, it's hard to identify before cell subset analysis. Immune cell types exhibited different distribution between metastasis and non‐metastasis groups (Figure 1C). DEGs between metastasis and non‐metastasis groups were analyzed. We found that immune system‐related genes such as LYZ, S100A6, TIMP1, S100A4, IGHG3, and SPP1 were upregulated in the metastasis group. Meanwhile, reduction of energy metabolism‐related genes such as CBR1, APOC1, and APOA2 were also found in the metastasis group (Figure 1D).

**Figure 2:**
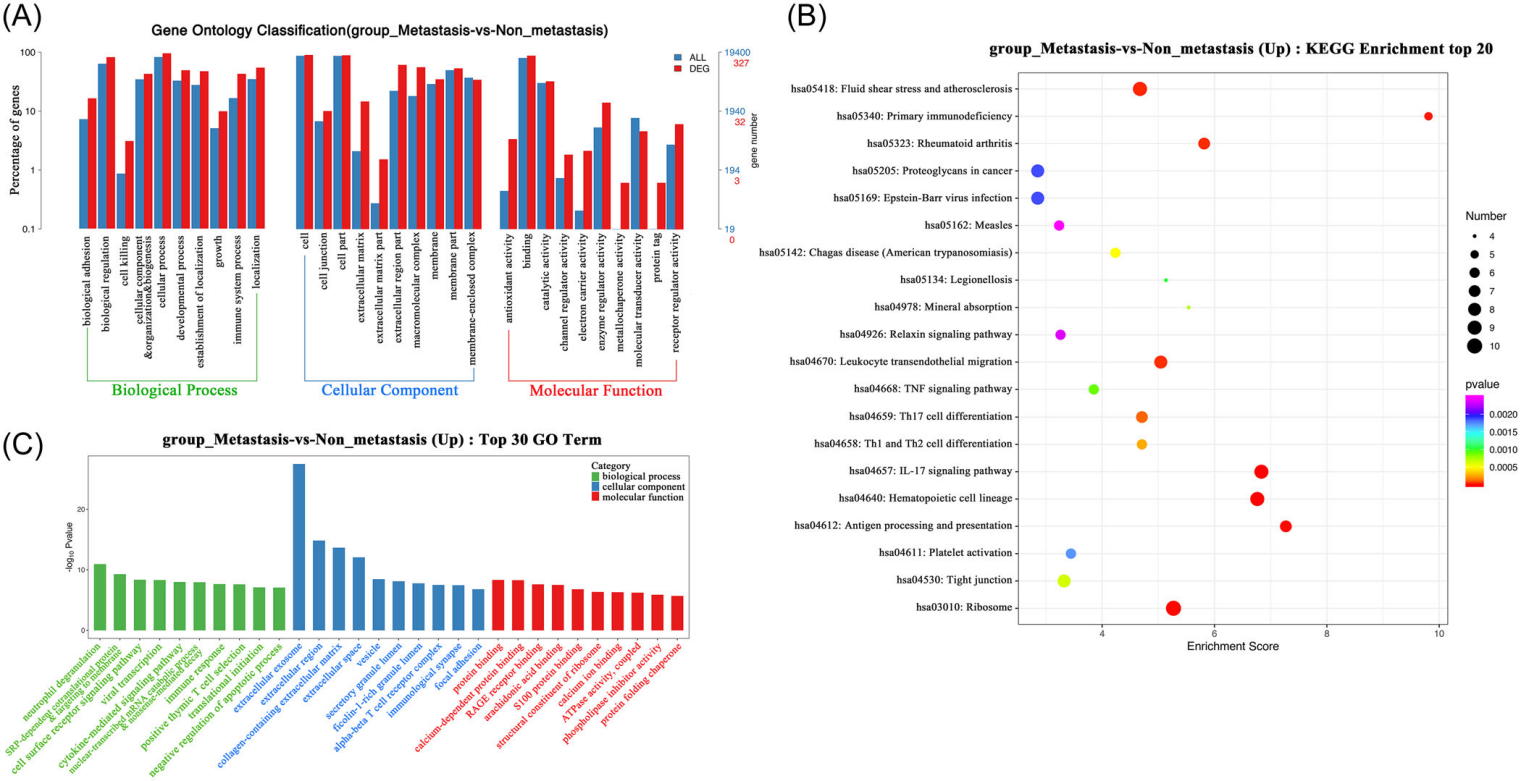
Functional enrichment of differentially expressed genes (DEGs) between metastasis and non‐metastasis group. (A) Go classification of total DEGs between the two groups. (B) KEGG pathway analysis of upregulated DEGs in metastasis group. (C) Go analysis of upregulated DEGs in metastasis group.

GO and KEGG pathway analysis of DEGs showed that DEGs mainly participate in biological processes such as biological adhesion, immune system process, and metabolism process (Figure 2A). As for upregulated DEGs in the metastasis group, KEGG pathway analysis showed that primary immunodeficiency, Th17 cell differentiation, IL‐17 signaling pathway, Th1 and Th2 cell differentiation, antigen processing, and presentation pathways were the main metastasis‐associated immune pathways (Figure 2B). Meanwhile, GO analysis showed that upregulated DEGs in the metastasis group mainly involved in biological processes such as cell surface receptor signaling pathway, cytokine‐mediated signaling pathway, immune response (Figure 2C).

### 3.2 Heterogeneity of NK/T cells and myeloid cells

**Figure 3:**
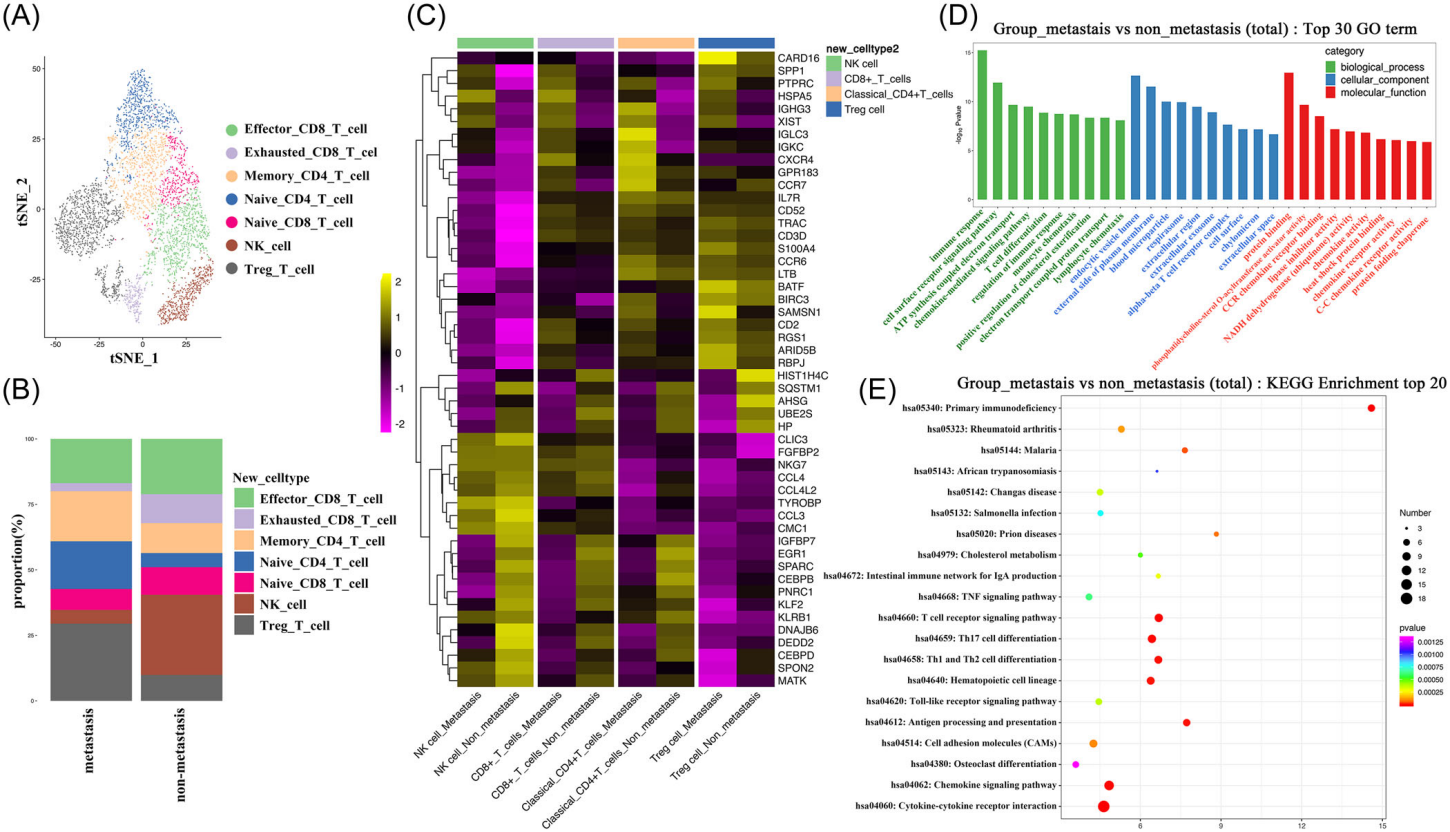
Heterogeneity of NK‐T cells between metastasis and non‐metastasis group. (A) NK‐T cell subtypes were identified, including effector CD8^+^ T cells, exhausted CD8^+^ T cells, memory CD4^+^ T cells, Naïve CD4^+^ T cells, Naïve CD8^+^ T cells, NK cells, and Tregs. (B) Composition of different NK‐T cell subtypes between metastasis and non‐metastasis group. (C) Differentially expressed genes (DEGs) of NK‐T cells between the two groups. (D) GO analysis of total DEGs on NK‐T cells between the two groups. (E) KEGG pathway analysis of total DEGs on NK‐T cells between the two groups.

To reveal the metastasis‐associated heterogeneity of NK/T cells and myeloid cells, functional subtypes were identified. (Figure 3A). Enrichment of Tregs and Naïve CD4+ T cells was found in the metastasis group (Figure 3B). Furthermore, DEGs of NK‐T cells between metastasis and non‐metastasis groups were analyzed, and differential expression genes related to T cell‐mediated cytotoxicity were found. For example, SPP1 was found to be upregulated in NK cell and CD8^+^ T cell. PTPRC was found to be upregulated in all NK/T subtypes (Figure 3C). GO analysis showed that DEGs of NK‐T cells most participate in biological processes such as T cell differentiation, and lymphocyte chemotaxis (Figure 3D), KEGG analysis showed DEGs of NK‐T cells most participate in primary immunodeficiency, T cell receptor signaling, Th17 cell differentiation, etc. (Figure 3E).

**Figure 4:**
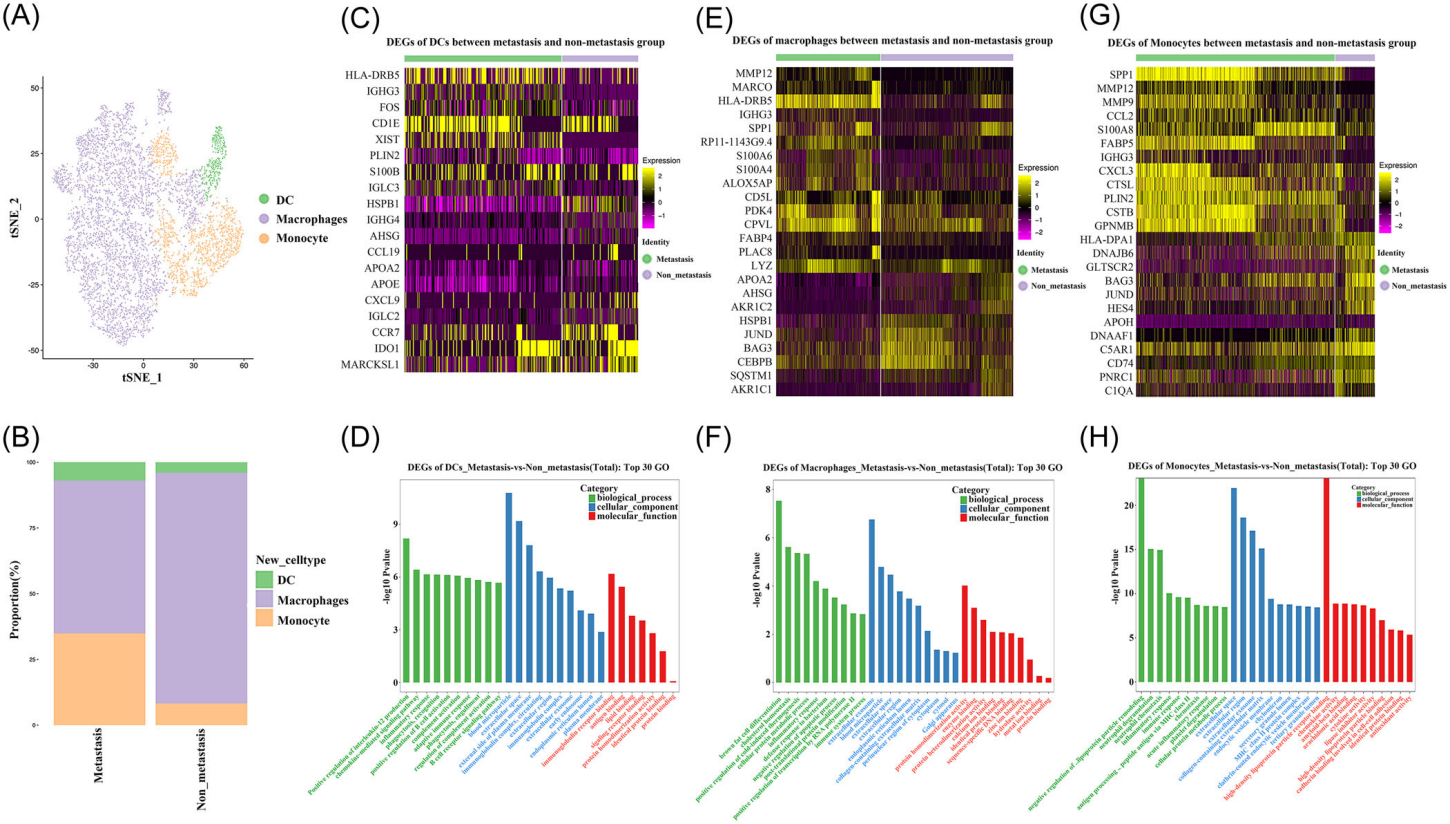
Heterogeneity of myeloid cells between metastasis and non‐metastasis group. (A) Myeloid cell subtypes were identified including DCs, macrophages, and monocytes. (B) Composition of different myeloid cell subtypes between metastasis and non‐metastasis group. (C) Differentially expressed genes (DEGs) of dentritic cells between the two groups. (D) GO of total DEGs on dentritic cells between the two groups. (E) DEGs of macrophages between the two groups. (F) GO of total DEGs on macrophages between the two groups. (G) DEGs of monocyte between the two groups. (H) GO of total DEGs on monocyte between the two groups. DCs, dendritic cells.

Among myeloid cells, DCs, macrophages, and monocytes were identified (Figure 4A). Significant enrichment of monocytes and reduction of macrophages were found in the metastasis group (Figure 4B). Among DEGs of DCs between metastasis and non‐metastasis groups, migration markers such as CCR7, CCL19, and CXCL9 were found to be upregulated in the non‐metastasis group (Figure 4C). GO analysis showed that metastasis‐related DEGs of DCs mainly involve in IL‐12 production, chemokine‐mediated signaling pathway, and inflammatory response (Figure 4D). In regard to DEGs of macrophages, immune suppressive genes such as SPP1, S100A4, and S100A6 were significantly upregulated in metastasis group (Figure 4E). GO and KEGG analysis showed that DEGs of macrophages mainly participated in cholesterol homeostasis, cellular protein metabolic process, and inflammatory response (Figure 4F). As for DEGs of monocyte, immune suppressive genes such as SPP1, S100A8, and CCL2 were significantly upregulated in the metastasis group (Figure 4G). GO and KEGG analysis showed that DEGs of monocyte mainly participated in neutrophil chemotaxis, immune response, antigen processing, and presentation (Figure 4H).

### 3.3 Tregs clustering and pseudotime analysis

**Figure 5:**
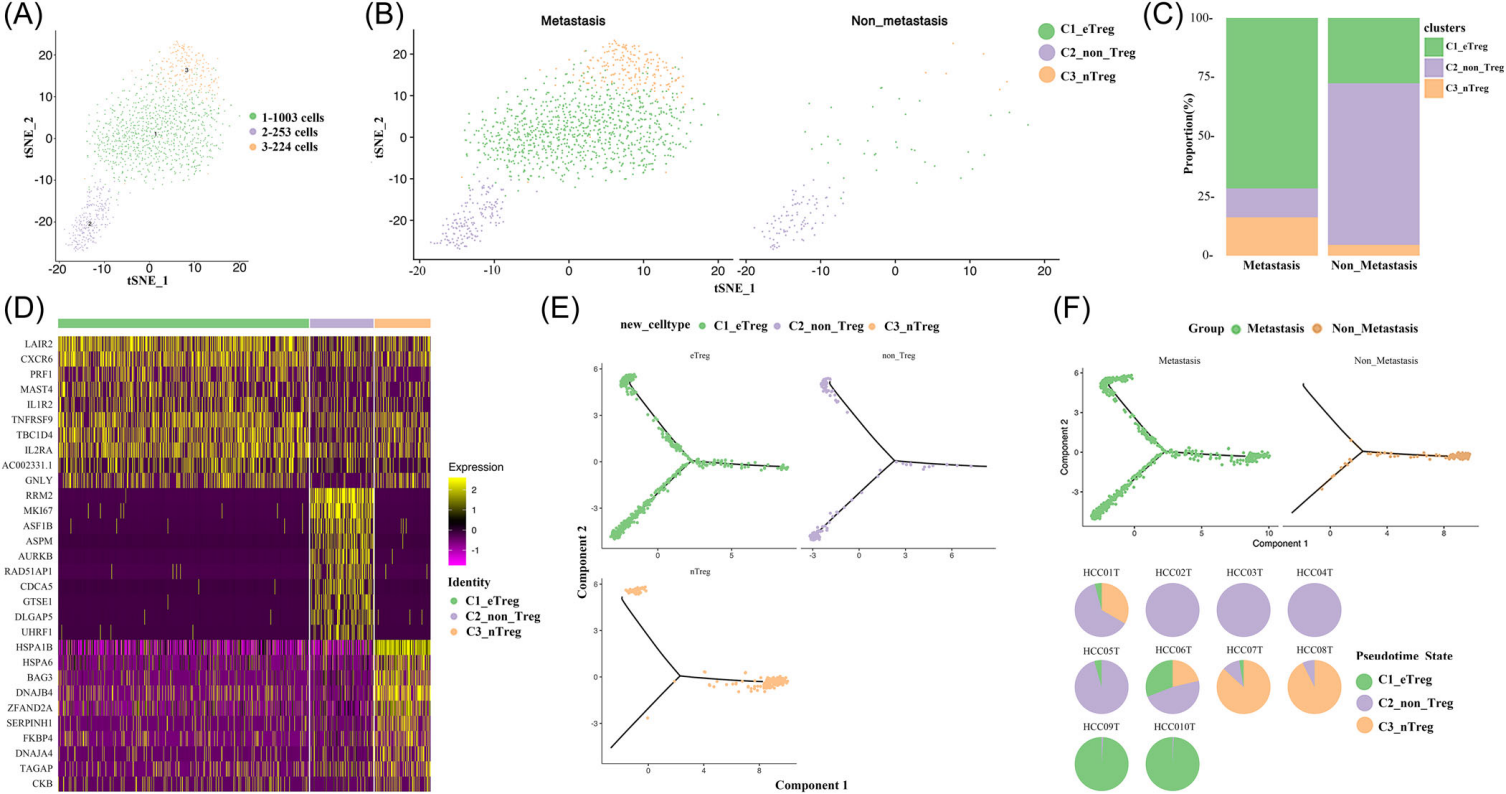
Clustering and pseudotime analysis of Tregs. (A) Three main clusters of Tregs were identified, and the gene expression characteristics of each cluster were different: C1_eTreg, C2_non‐Treg, and C3_nTreg. (B) The t‐SNE projection of Treg cell clusters between metastasis and non‐metastasis group. (C) Composition of different Treg cell clusters between the two groups. (D) Top 10 marker genes of each Treg cell cluster. (E) Pseudotime analysis of trajectory evolution of different Treg cell clusters. (F) Trajectory states of different Treg cell clusters between the two groups and in each HCC patient. HCC, hepatocellular carcinoma.

Significant enrichment of Tregs in the metastasis group was found (41.16% vs 22.26%, *p* = .048, Figure 2B). Thus, functional subtypes of Tregs were further revealed. A total of three clusters of Tregs were identified including C1_Treg‐FOXP3, C2_Treg‐STMN1, and C3_Treg‐HSPA1B (Figure 5A). Enrichment of C1_Treg‐FOXP3 and reduction of C2_Treg‐STMN1 were found in metastasis group (Figure 5B,C). C1_Treg‐FOXP3 cluster cells were characterized by the expression of FOXP3, CTLA‐4, and could be defined as effector Tregs (eTregs). C2_Treg‐STMN1 cluster cells could be defined as natural Tregs (nTregs). C3_Treg‐HSPA1B cluster cells were characterized by low expression of CD25 and be defined as non‐Treg (Figure 5D). Moreover, Pseudotime analysis showed that the pseudotime process began with nTreg and ended with eTreg and non‐Treg (Figure 5E). From the non‐metastasis to metastasis state, enhanced differentiation of eTreg occurred, which suggested an important role of eTreg in advanced HCC. Specifically, eTreg cluster cells were dominant Tregs in 9th and 10th HCC patients who were at stage Ⅳ while nTreg cluster cells were dominant Tregs in HCC patients at early/intermediate stage (Figure 5F).

### 3.4 Identification and confirmation of exhaustion genes of Tregs

**Figure 6:**
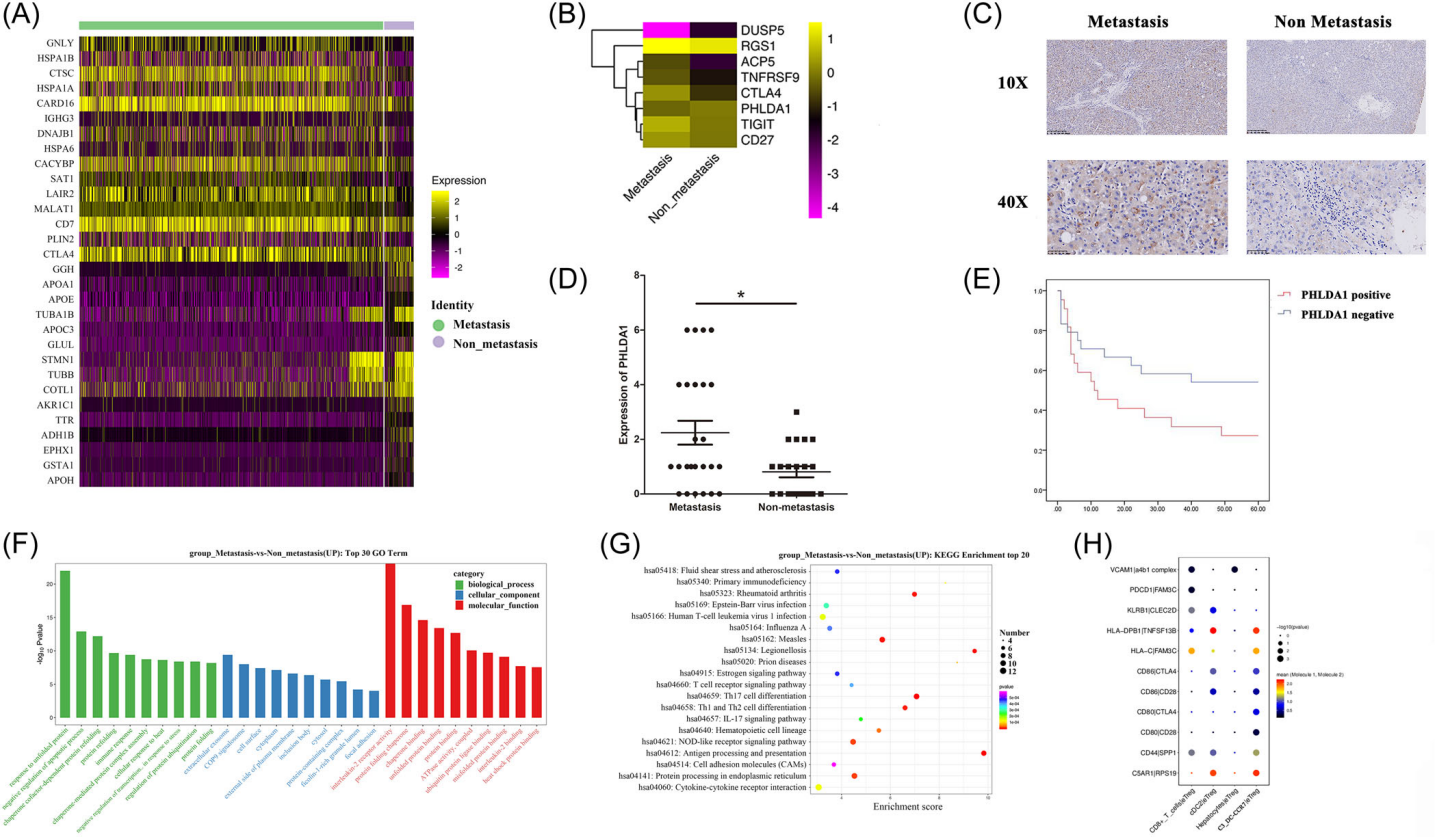
Gene expression characterization of Tregs. (A) Differentially expressed genes (DEGs) of Tregs between metastasis and non‐metastasis group. (B) Expression of common genes of exhausted CD8^+^ T cells and Tregs between the two groups. (C) Representative results of immunohistochemical staining of PHLDA1 in 46 HCC tissues. (D) Mean scores (Mean ± SD) of immunohistochemical staining intensity of PHLDA1 in hepatocellular carcinoma (HCC) tissues with metastasis (*n* = 25) and without metastasis (*n* = 21), *p* = .008, t‐test (two independent samples). (E) Kaplan–Meier survival curves of PHLDA1 positive (*n* = 26) and negative (*n* = 20) HCC patients based on 5‐year overall survival data, *p* = .045, log‐rank chi‐square = 4.032; Kaplan–Meier survival curves of metastasis group (*n* = 25) and negative (*n* = 21) HCC patients based on 5‐year overall survival data, *p* = .001, log‐rank chi‐square = 11.34. (F) GO analysis of upregulated DEGs of Tregs in metastasis group. (G) KEGG pathway analysis of upregulated DEGs of Tregs in metastasis group. (H) Interaction analysis among eTreg, hepatocytes, CD8^+^ T cells, and DCs. The gradation from black to red means a stronger interaction strength. The diameter of the circle means a significance based on the probability on specific ligand/receptor interaction. DCs, dendritic cells.

To further explore the potential exhaustion marker gene of Tregs, we analyzed and validated DEGs of Tregs between metastasis and non‐metastasis groups (Figure 6A). The expressions of previously reported common genes of CD8^+^ T cells and Tregs were analyzed, and results showed that CTLA4, ACP5, TIGHT, RGS1, TNFRSF9, PHLDA1, CD27, and DUSP4 were significantly upregulated in metastasis group (Figure 6B). Among them, the prognostic value of RGS1, PHLDA1, and DUSP4 has not been validated in HCC tissues in previous studies. Thus, we conducted preliminary IHC of RGS1, PHLDA1, and DUSP4 using 3 HCC tissues and 3 HCC adjacent tissues. Results showed that PHLDA1 was highly expressed in HCC tissues. Furthermore, we examined the expression of PHLDA1 in HCC tissues derived from 46 patients in Sichuan Cancer Hospital and got informed consent from each patient. Results showed that the expression of PHLDA1 was significantly higher in patients with metastasis (Figure 6C). The mean expression of PHLDA1 was 2.24 in the metastasis group and 0.81 in the non‐metastasis group (*p* < .05) (Figure 6D). Kaplan–Meier survival analysis showed that PHLDA1 positive patients had poor prognosis (log‐rank chi‐square = 4.032, *p* = .045). Meanwhile, Kaplan–Meier survival results also showed that HCC patients with metastasis had poor prognosis (log‐rank chi‐square = 11.34, *p* = .001) (Figure 6E). Therefore, expression of PHLDA1 was associated with metastasis and prognosis. GO and KEGG analyses were conducted based on DEGs of Tregs between the metastasis and non‐metastasis groups. Upregulated DEGs in the metastasis group mainly regulated the apoptotic process (Figure 6F). Tregs showed extensive immune regulation effects on Th17 cells, Th1 cells, Th2 cells differentiation and antigen processing and presentation pathway et al. (Figure 6G). To investigate the interaction network among Tregs, CD8^+^ T cells, and DCs, we used a set of immune‐related ligand–receptor to conduct cell–cell communication analysis. Results showed that Tregs could bind to PDCD1 and regulate the cell cycle of CD8^+^ T cells; also, Tregs could bind to mature makers such as CD80 and CD86 of DCs and influence the antigen processing and presentation (Figure 6H).

### 3.5 DCs clustering, pseudotime analysis

**Figure 7:**
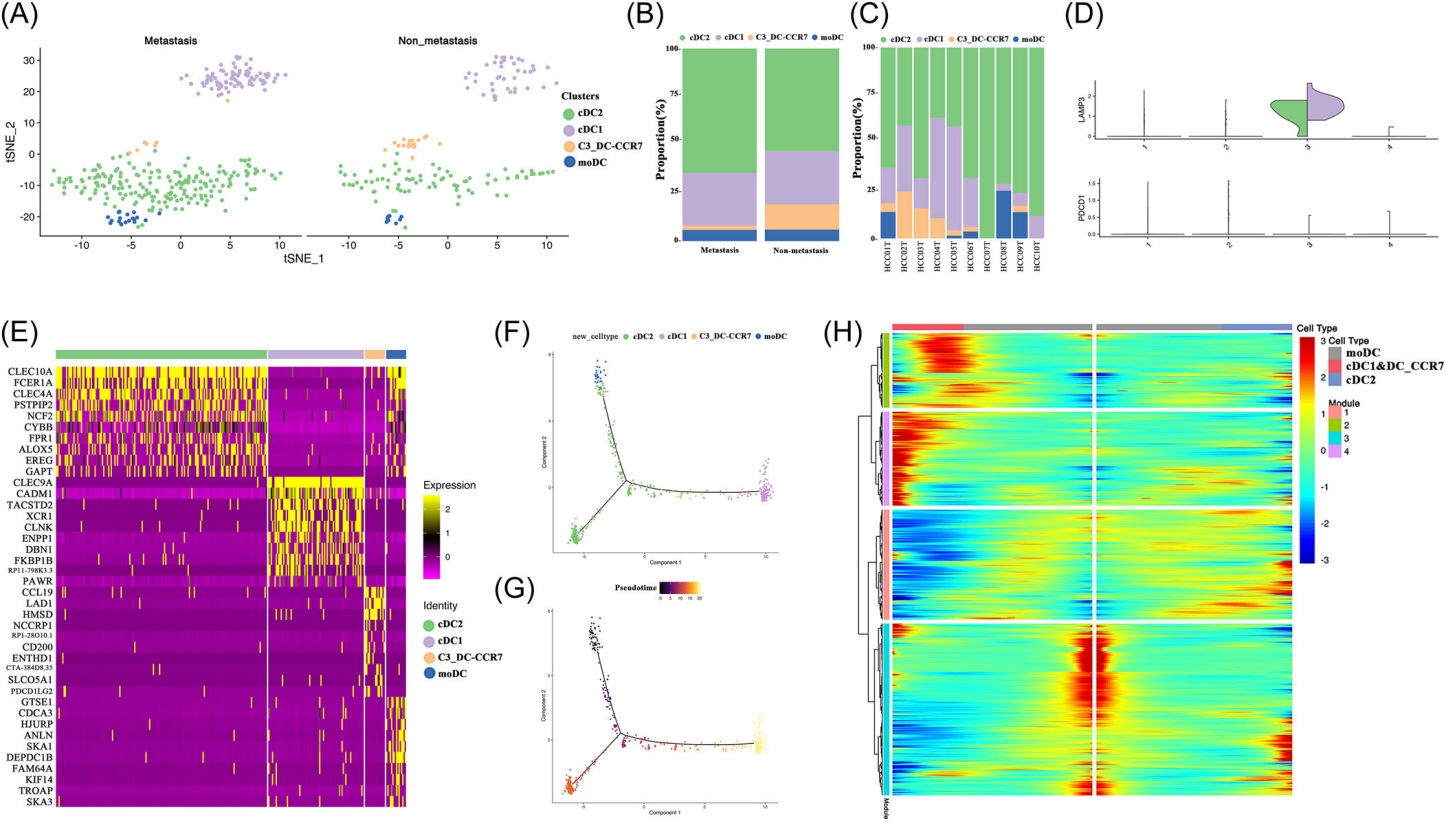
Clustering and pseudotime analysis of dendritic cells (DCs). (A) The t‐SNE projection of DC cell clusters between metastasis and non‐metastasis groups shows four main clusters of DCs with distinct gene characteristics: C1_cDC2, C2_cDC1, C3_DC‐CCR7, and C4_moDC. (B) Composition of different DCs clusters between the two groups. (C) Composition of different DCs clusters in each HCC patient. (D) Violin plot of LAMP3 and PDCD1 expression in each DC cell cluster. (E) Top 10 marker genes of each DC cell cluster. (F) Trajectory evolution of different DC cell clusters. (G) Trajectory state evolution of different DC cell clusters. (H) Heat map of genes expression in different pseudotime state. HCC, hepatocellular carcinoma.

Four clusters of DCs were identified, including C1_DC‐CLEC10A, C2_DC‐CLEC9A, C3_DC‐CCR7, and C4_DC‐ITGAM (Figure 7A). Among them, C3_DC‐CCR7 was a novel DC cell cluster and significantly enriched in the non‐metastasis group (10.11% vs 0.86%, *p* = .045) (Figure 7B,C). LAMP3 was previously reported to be associated with DC maturation. In our data, we also found that LAMP3 rather than PDCD1 was associated with metastasis (Figure 7D). C1_DC‐CLEC10A was characterized by high expression of CLEC10A, FCER1A, and CD1C, representing cDC2. C2_DC‐CLEC9A was characterized by high expression of CLEC9A, XCR1, and CADM1, representing cDC1. C3_DC‐CCR7 was characterized by high expression of CCR7, CCL19, LAMP3, and CD83, representing a novel cluster. C4_DC‐ITGAM was characterized by high expression of STMN1, ITGAM, and CCL3, representing Mono DC (Figure 7E). We next examined the trajectory of DC cell clusters; C3_DC‐CCR7 seemed to be matured from mono DC and differentiated to cDC1 (Figure 7F,G). Moreover, although C3_DC‐CCR7 and cDC1 showed similar pseudotime state, it's notable that the heat map of gene expression in different pseudotime state revealed distinct genes of C3_DC‐CCR7 such as CCR7, CCL19 when compared with cDC1 (Figure 7H).

### 3.6 Interaction analysis of C3_DC‐CCR7 cell cluster

**Figure 8:**
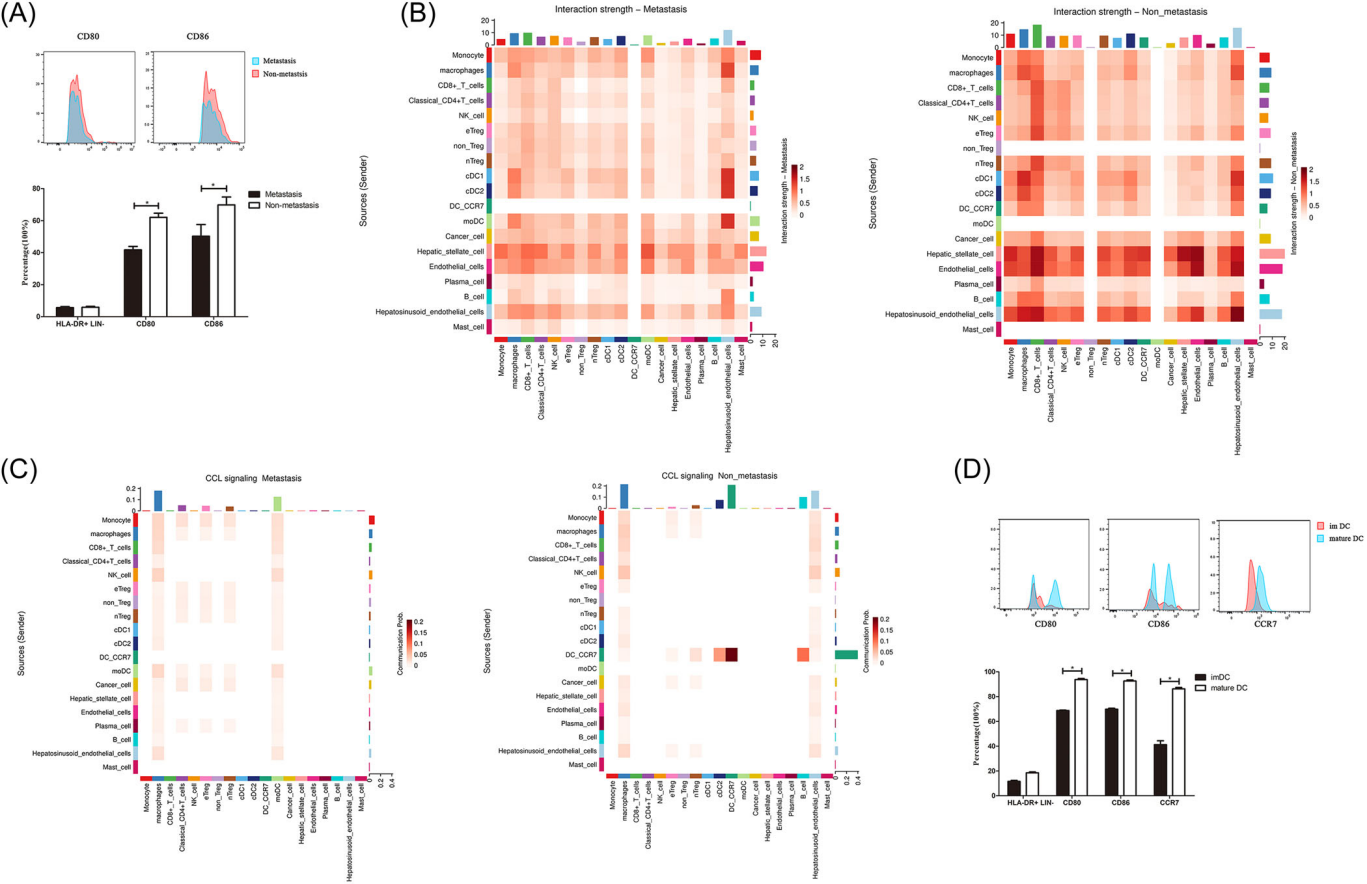
Interaction analysis of dendritic cells (DCs). (A) Tumor‐infiltrating lymphocytes from hepatocellular carcinoma (HCC) patients with metastasis (*n* = 3) and without metastasis (*n* = 3) were sorted out, and maturation of DCs was validated by flow cytometry, mean percentage (Mean ± SD) of HLADR+LIN− (*p* = .77), CD80 (*p* = .004)* and CD86 (*p* = .021) * were compared via *t*‐test (two independent samples). (B) Interaction strength heat map among immune cells between metastasis and non‐metastasis group. (C) Interaction strength of CCR7/CCL19 signaling between metastasis and non‐metastasis group. (D) Flow cytometry examination of maturation of DCs after in vitro culture of DCs derived from peripheral blood of three HCC patients. Mean expression (Mean ± SD) of HLA‐DR + LIN‐ (*p* = .22), CD80 (*p* = .001), CD86 (*p* = .000), and CCR7 (*p* = .003) in immature DCs and mature cells after in vitro culture were compared via *t*‐test (paired samples).

To validate the inhibition of the maturation of DCs in HCC patients with metastasis, we selected 3 tumor tissues from HCC patients with metastasis and 3 tumor tissues from HCC patients without metastasis. CD45^+^ positive lymphocytes were sorted out using magnetic beads. Flow cytometry was conducted to examine the maturation of DCs. Results showed that the expression of CD80 (41.77% vs 62.03%, *p* < .05) and CD86 (50.27% vs 69.8%, *p* < .05) was significantly higher in a non‐metastasis group than metastasis group. The expression of HLA‐DR was similar between the two groups (Figure 8A). To investigate the interactions between C3_DC‐CCR7 and other lymphocytes, we conducted cell–cell communication analysis. Results showed that interaction strength between C3_DC‐CCR7 rather than cDC1 with other immune cells was significantly reduced in the metastasis group (Figure 8B). Specifically, C3_DC‐CCR7 interacted with macrophages, eTregs, nTregs, cDC2 via CCR7‐CCL19 signaling (Figure 8C). To verify the association between CCR7 and with maturation of DCs. In vitro culture and inducement of maturation of DCs were conducted. We selected peripheral blood samples from three HCC patients with metastasis. DCs were cultured and induced to maturation. At last, the expression of mature genes and CCR7 were tested by flow cytometry. Results showed that CD80 (68.67% vs 93.67, *p* < .05), CD86 (69.8% vs 92.53%, *p* < .05), CCR7 (41.13% vs 86.2%, *p* < .05) were significantly upregulated after maturation of DCs in vitro (Figure 8D).

## 4 DISCUSSION

HCC tumor immune microenvironment was reported to be classified into five major subtypes of immune microenvironment of HCC including immune activation, immune suppressive myeloid, immune suppressive stromal, immune exclusion, and immune residence in a large cohort of single‐cell sequencing research.^16^ In the present study, the HCC metastasis‐related immune microenvironment generally exhibited distinct signatures including enrichment of Tregs and restricted maturation of DCs, which was similar to the immune suppressive myeloid subtype. Concretely, immunosuppression‐related signature genes such as SPP1 and S100A8 were upregulated DEGs in metastasis groups. Secreted Phosphoprotein 1 (SPP1) was reported to be highly expressed in TAMs in tumor diseases^18^, ^19^ and related to less efficacy of anti‐PD‐L1 therapy,^20^ S100A8 was reported to be associated with tumorigenesis and poor differentiation.^21^, ^22^ On the contrary, upregulation of immune activation associated genes like LAMP3, KLRB1, and APOA2 was also found in HCC without metastasis. LAMP3 was associated with DC maturation and migration.^23^, ^24^ KLRB1 is associated with a better prognosis in most types of cancer.^25^ APOA2 was related to lipid metabolism which was important in immune cell development.^26^ These results revealed that HCC with metastasis exhibited more suppressive immune microenvironment than HCC without metastasis.

Tregs represented the main component of suppressive immune cells. In our study, we found that enrichment of effector Tregs rather than exhausted CD8^+^ T cells was the major immune signature of HCC with metastasis. For the reason that direct depletion of Tregs may induce aberrant autoimmunity,^27^ exhausted genes both expressed in Tregs and exhausted CD8^+^ T cells could be promising targets of ICIs. In our study, exhausted genes such as CTLA4, TIGIT, and PHLDA1 were found to be upregulated in Tregs in HCC with metastasis. Moreover, we found that PHLDA1 was significantly associated with poor prognosis, which was similar with results in previous research using TCGA cohort gene expression data.^11^, ^28^ CTLA4 and TIGIT have demonstrated their immune suppressive roles in clinical.^29^, ^30^ Recently, PHLDA1 was reported to be involved in programmed cell death and regulated tumorigenesis, metastasis, cell proliferation, apoptosis.^31^ As for immunity, PHLDA1 was first identified as the exhaustion gene of Tregs in colon and lung cancer.^32^ Expression of PHLDA1 could decrease the infiltrating of T cells.^33^ Therefore, PHLDA1 is a unique maker gene of Tregs and could be a promising target for immunotherapy.

DCs play the predominant role in tumor antigen presentation and immune activation. DCs could be divided into three major subsets: plasmacytoid DC, conventional DC 1 (cDC1), and conventional DC 2 (cDC2).^34^ Meanwhile, monocyte‐derived DC (mo‐DC) was similar with cDC2 in some settings. The maturation of DCs was necessary for T‐cell trafficking and activation.^35^ In our study, we identified a novel subset of mature DCs with high expression of maturation markers CD80, CD86, and LAMP3, and migration markers CCR7 and CCL19. Pseudotime analysis showed that inhibition of differentiation occurred in CCR7^+^ DCs rather than cDC1 in HCC with metastasis. Interaction analysis also showed that CCR7^+^ DCs had profound regulatory effects on immune cells. CCR7 was reported to be one of the migration markers of immune cells in previous studies,^36^ which determines the pathogen or antigen presentation.^37^ Expression of CCR7 in DCs was also demonstrated in live images in melanoma and associated with a better prognosis.^38^, ^39^ CCL19 was a ligand of CCR7. In a previous study, CCL19 was also reported to be associated with infiltration of immune cells.^40^ Considering that CCR7 agonist was reported to increase antitumor immune activity^41^ and downregulate expression of exhaustion markers including PD‐1 and TIGIT.^42^ We supposed that CCR7 may be a promising target in DC cell‐based immune cell therapy.

In conclusion, our study revealed the metastasis‐associated immune signatures via landscaping the heterogeneity of immune cells in HCC at different stages. Enriched infiltration of eTregs and impaired differentiation of CCR7^+^ DCs were the main characteristics in HCC with metastasis. Our data provide evidence that PHLDA1 could be a novel therapeutic target of ICIs to reduce the infiltration of Tregs. Moreover, CCR7 may be a promising target to promote the maturation and migration of DCs in HCC.

## AUTHOR CONTRIBUTIONS

**Deyuan Zhong**: Methodology; software; validation; writing—original draft. **Ying Shi**: Conceptualization; project administration; supervision; writing—review & editing. **Wenzhe Ma**: Data curation; methodology; resources; validation; writing—review & editing. **Yuxin Liang**: Methodology; software; validation; visualization. **Hanjie Liu**: Methodology; software; validation; visualization. **Yingying Qin**: Methodology; software; validation; visualization. **Qinyan Yang**: Methodology; resources; validation; visualization. **Xiaolun Huang**: Conceptualization; project administration; resources; supervision; writing—review & editing. **Yuanjun Lu**: Conceptualization; data curation; project administration; supervision; validation; visualization; writing—review & editing. **Jin Shang**: Conceptualization; data curation; methodology; project administration; resources; software; writing—original draft.

## CONFLICT OF INTEREST STATEMENT

The authors declare no conflicts of interest.

## ETHICS STATEMENT

This study was approved by the ethics committee of Sichuan Cancer Hospital and was conducted according to the 1975 Declaration of Helsinki. Informed consent was obtained from each patient whose medical records were analyzed.

## Supporting information

Supporting information.

Supporting information.

Supporting information.

## Data Availability

The data used to support the findings of this study are available from the corresponding author upon reasonable request.
